# 3DMolNavi: A web-based retrieval and navigation tool for flexible molecular shape comparison

**DOI:** 10.1186/1471-2105-13-95

**Published:** 2012-05-14

**Authors:** Yu-Shen Liu, Meng Wang, Jean-Claude Paul, Karthik Ramani

**Affiliations:** 1 School of Software, Tsinghua University, Beijing 100084, China; 2 Key Laboratory for Information System Security, Ministry of Education of China, Beijing 100084, China; 3 Tsinghua National Laboratory for Information Science and Technology, , Beijing 100084, China; 4 INRIA, Domaine de Voluceau-Rocquencourt, , 78153 Le Chesnay Cedex, France; 5 School of Mechanical Engineering, Purdue University, West Lafayette, IN, 47907, USA

## Abstract

**Background:**

Many molecules of interest are flexible and undergo significant shape deformation as part of their function, but most existing methods of molecular shape comparison treat them as rigid shapes, which may lead to incorrect measure of the shape similarity of flexible molecules. Currently, there still is a limited effort in retrieval and navigation for flexible molecular shape comparison, which would improve data retrieval by helping users locate the desirable molecule in a convenient way.

**Results:**

To address this issue, we develop a web-based retrieval and navigation tool, named 3DMolNavi, for flexible molecular shape comparison. This tool is based on the histogram of Inner Distance Shape Signature (IDSS) for fast retrieving molecules that are similar to a query molecule, and uses dimensionality reduction to navigate the retrieved results in 2D and 3D spaces. We tested 3DMolNavi in the Database of Macromolecular Movements (MolMovDB) and CATH. Compared to other shape descriptors, it achieves good performance and retrieval results for different classes of flexible molecules.

**Conclusions:**

The advantages of 3DMolNavi, over other existing softwares, are to integrate retrieval for flexible molecular shape comparison and enhance navigation for user’s interaction. 3DMolNavi can be accessed via https://engineering.purdue.edu/PRECISE/3dmolnavi/index.html.

## Background

The geometrical shape of a molecule has been widely acknowledged as a key factor for biological activity and thus is regarded as a very important pattern for which to search in various applications, such as computer aided molecular design, rational drug design, molecular docking and function prediction. For example, a newly discovered protein is predicted to exert the same function as the most similar proteins in a database with known proteins. This similarity among two proteins can be defined in many different ways, such as their sequences aligning well, or structures matching well, or geometrical shape matching well, or both having common surface clefts or bindings sites, or similar chemical features [1-3]. To exploit the geometrical shape similarity between molecules, a useful tool is *molecular shape comparison* that compares the shapes of two or more molecules and identifies common spatial features [2,3]. The underlying assumption is that molecules similar to the active query molecule are likely to share the similarly spatial properties. When using molecular shape comparison, the molecules with shape similarity can be found without any specification of chemical structures.

Although many researchers have proposed various methods for molecular shape comparison [2-6], most of them treat molecules as rigid shapes and only few attentions are paid on the deformed shapes of flexible molecules. Nevertheless, many molecules of interest are flexible and often undergo significant shape deformation as part of their function. When flexible molecules in different conformations are compared to each other as rigid bodies, strong shape similarities may be missed. Methods of molecular shape comparison can be operated with either global or local matching methods. Most existing global rigid matching methods [2-6] are only effective for comparing 3D rigid objects, but they cannot handle the deformed shapes of flexible objects well. Alternatively, if one performs local alignment and considers multiple solutions from local structure alignments [7,8], many conformational changes can be effectively detected. However, many times, the local matching methods do not provide a good description of the overall shape.

To address this issue, our recent studies have presented several global matching methods for flexible shapes based on a new shape intrinsic measure, called Inner Distance Shape Signature (IDSS) [9-11]. Compared with the traditional structural alignment methods, our methods only considers the geometrical shape of a molecule without requiring any prior structural alignment and any specification of chemical structure. In contrast to local matching methods, our methods does not require any detection for prior knowledge of the flexible regions. In our new shape signature, the inner distance is defined as the length of the shortest path between landmark points within the molecular shape. We found that the inner distance reflects well the molecular structure and deformation without explicit decomposition. The IDSS is stored as a histogram which is a probability distribution of inner distances between all sample point pairs on the molecular surface. The IDSS is insensitive to shape deformation of flexible molecules and more effective at capturing molecular structures than traditional molecular shape comparison methods. In particular, the point is that our approach reduces the 3D shape comparison problem of flexible molecules to the comparison of IDSS histograms, where the signatures are deformation-invariant, making it robust and efficient. We also presented many examples, both of detailed pairwise comparisons and of database, using the flexible database of motions.

In this paper, we develop a new web-based retrieval and navigation tool, named 3DMolNavi, for flexible molecular shape comparison. This tool aims to fast search the database of flexible molecules by combining the IDSS histogram, and it also supports the intuitive navigation for the searched results in 2D and 3D spaces by combing dimensionality reduction techniques.

### Related work

The methods of molecular shape comparison can be roughly divided into two categories [2,9,10]: superposition methods and descriptor methods. The former relies on finding an optimal superposition/alignment of two or more molecules compared, and the later (i.e. non-superposition) is independent of molecular orientation and position.

#### Superposition methods

The superposition methods usually compare molecular shapes in a particular coordinate system by a priori superposition/alignment, which is non-trivial to achieve robustly. The representative superposition methods, for example, are based on protein structure comparison. There have been quite a few papers in the area of flexible protein structure comparison and some papers using atom-atom distances for structure comparison. A widely used strategy is based on the root-mean-square deviation (RMSD) measure of the average distance between the atoms (usually the backbone atoms) of superimposed proteins. For instance, Damm and Carlson [12] developed a structural comparison method for flexible proteins using a Gaussian-weighted RMSD fit, which calculates the minimal weighted deviation between the two coordinate sets. In order to overcome the large displacement of flexible proteins, we recently presented a new structural comparison method for flexible proteins between their atomic coordinates using least median of squares [13]. Theobald et al. [14,15] proposed and applied the principle of maximum likelihood to macromolecular structure comparison by assuming a Gaussian distribution of the whole structures in the analysis. In addition, several methods have been developed for flexible protein structure comparison, which automatically identifies hinges and internal rearrangements in two protein structures [7,8]. The reader may consult Refs. [7,8,12,13] for a review of some available methods for flexible protein structure comparison using atom-atom distances.

However, most of flexible protein structure comparison methods are based on structural alignment algorithms. Structural alignment aims to compare a pair of structures, where the alignment between equivalent residues is not given prior. Therefore, an optimal sequence alignment needs to be identified, which has been shown to be NP-complete [16]. The tool developed in the paper is simpler than the traditional structural alignment methods, it does not require any prior structural alignment and has no any specification of chemical structure.

#### Descriptor methods

The descriptor methods are independent of molecular orientation and position by using descriptor to represent the shape of molecule. The descriptor methods compute the similarity score by comparing the corresponding descriptors between two molecular shapes without any superposition. A shape descriptor, or named signature, is a compact representation for some essence of the shape.

An early molecular shape description is developed by Bemis et al. [17] by considering each molecule as a collection of its 3-atom submolecules. Nilakantan et al. [18] also introduced a method for the rapid quantitative shape matching between two molecules or a molecule and a template, using atom triplets as descriptors. Several recent works have been developed for molecular shape comparison in various definition of shape descriptors, including shape distribution descriptor, spherical harmonic signature, 3D Zernike descriptor, LightField shape descriptor, moment invariant descriptor, etc [4-6,19-25]. These descriptors are rigid-body-transformation invariant, and they are effective for matching rigid objects. Nevertheless, none of these methods is deformation invariant and they can not support flexible molecular shape comparison.

The shape descriptor is often used as an index in a database of shapes and it enables fast queries and retrieval. The descriptor methods are simpler and much faster than the superposition methods in searching large molecular databases. The shape descriptor is often represented by a histogram or vector. The IDSS histogram [9-11] also belongs to descriptor methods. A review of many available methods for molecular shape comparison is beyond the scope of this paper. The reader may consult Ref. [2,9,10] for detailed expositions.

By combining shape histograms, some related studies have been developed towards the retrieval and navigation tools that enhance user’s interaction and comprehension for molecular shape comparison. For instance, Sael et al. [6] developed a web-based 3D-SURFER software (http://dragon.bio.purdue.edu/3d-surfer/) for protein surface retrieval based on histogram of 3D Zernike moments. 3D-SURFER treats molecules as rigid shapes for searching, however, can not handle the flexible molecules. In addition, 3D-SURFER does not provide a navigation method for assisting the retrieval visualization and mining. Another similar tool was presented by [24] using the LightField shape descriptors (http://3d.csie.ntu.edu.tw/ProteinRetrieval/), which has the similar limitation to [6]. Recently, Pu et al. [26] implemented a software for retrieving 3D mechanical models and navigating the retrieved results in 2D and 3D spaces, but it is only for 3D mechanical models not for molecular shapes. In summary, there still is a limited effort in retrieval and navigation for flexible molecular shape comparison, which would improve data retrieval by helping users locate the desirable molecule in a convenient way.

In contrast to the previous work, we introduce a new web-based retrieval and navigation tool that can have better cognitive performance with concern to flexible molecular shapes by combining our previous methods [9,11]. We use this retrieval and navigation tool to enhance user’s interaction, better retrieval and visual data mining performance.

## Implementation

A molecule can be defined by a set of spherical atoms whose exposed surface represents a molecular surface that defines the boundary of the molecular volume. Here we consider the input molecule as a volumetric representation that is popularly used in many biological research fields. The volumetric model is built through first computing the Connolly surface of the molecule and then voxelizing it into a uniform 3D lattice.

Our retrieval system is based on the IDSS histogram for describing 3D shapes of flexible molecules, where IDSS is used as an index in a database of molecules and enables fast queries and retrieval. The original program [9] is available from https://engineering.purdue.edu/PRECISE/IDSS. One new version [11] for fast IDSS computation with advanced analysis and applications is at https://engineering.purdue.edu/PRECISE/VMID. The IDSSalgorithm is summarized as follows. 

· First, some point pairs are uniformly sampled on the molecular boundary surface using Lloyd’s algorithm.

· Then, inner distances of all sample point pairs are calculated by applying a shortest path algorithm.

· Finally, IDSS is built as the histogram/vector of values of inner distances using 128 bins.

**Figure 1:**
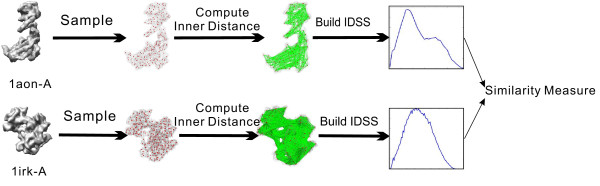
**The illustration of flexible molecular shape comparison and similarity measure based on the IDSS histograms.** Given a molecular shape, the whole process could be divided into three independent steps including sampling (red points) on the molecule, computing inner distances (green line segments) between all sample point pairs, and building the IDSS histogram. Consequently, our approach reduces the similarity measure among molecules to the comparison of IDSS histograms. An example is given for calculating the similarity measure between two different molecules (PDB codes: 1aon-A, 1irk-A), where the similarity score is equal to 0.6535.

**Figure 2:**
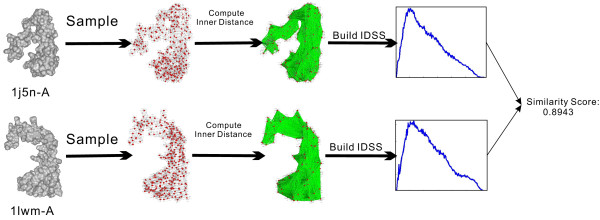
**Illustration of flexible molecular shape comparison and similarity measure between two conformations (PDB codes: 1j5n-A, 1lwm-A) of the same protein based on their IDSS histograms.** The IDSS is not sensitive to shape deformation, and consequently, two conformations have a high similarity score equal to 0.8943.

IDSS, being an intrinsic property, remains invariance under geometrical shape deformation of flexible molecules. The details of IDSS algorithm and implementation can be found at [9,11]. The similarity score between two histograms in our algorithm is calculated using the well-known Minkowski *L*_1_ norm of the Probability Density Functions (PDF) [22]. Figure 1 illustrates the flowchart of flexible molecular shape comparison and similarity measure based on the computed IDSS histograms, where two different molecules (PDB codes: 1aon-A, 1irk-A) have a low similarity score equal to 0.6535. Figure 2 gives an example of the similarity score between two conformations (PDB codes: 1j5n-A, 1lwm-A) of the same protein based on their IDSS histograms, where two conformations have a high similarity score equal to 0.8943.

Then we implement the web-based retrieval and navigation system by combing the above IDSS histogram for retrieval and Multidimensional Scaling (MDS) [27] for dimensionality reduction. Alternatively, some other dimensionality reduction techniques can also be used instead of MDS. Here, the MDS technique embeds the IDSS histogram/vector of each molecular shape into 2D and 3D spaces for the visualized navigation. The MDS algorithm and the visualized navigation are implemented by Matlab and Flex, respectively. In order to achieve fast interaction and visualization, we alternatively display the 2D image of each 3D molecular shape. In order to speed up the visualization during navigation, we set the “visibility” property to avoid loading too much images at one time. Any images within a certain controlled scope will be loaded and displayed, and others too far away from the view point would not be displayed. In addition, the molecules belonging to different classes are highlighted in different colors for having a better cognitive performance.

## Results

**Figure 3:**
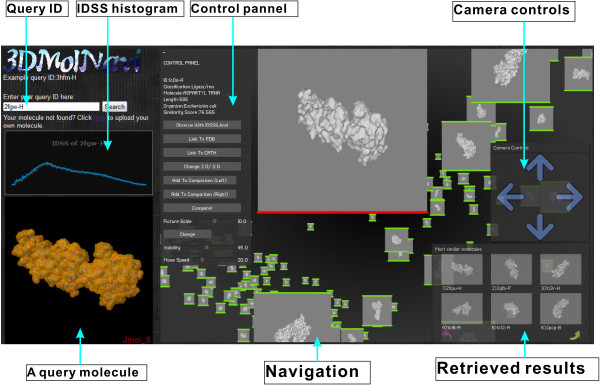
The 3DMolNavi graphical interface.

Figure 3 shows the 3DMolNavi interface, which consists of six parts. First, one specifies a protein ID as an input to a query on the top-left. Then 3DMolNavi will display the query 3D molecule shape using JMOL and the IDSS histogram on the left, and the retrieved results are shown on the bottom-right. The navigation of retrieved results is visualized on the right on a Flash platform. In the navigation viewer, the “Camera Controls” and “Control Panel” include some specific options. A comparative view for IDSS between two molecules is provided by clicking the “Compare!” button. Automatic and free navigation is supported here, that is, to travel to the molecule chosen automatically, and to move the camera freely to wherever one wants to go.

3DMolNavi is currently built on two databases. One database is from the CATH (http://www.cathdb.info/), which contains thousands of protein chains. The users can either search by PDB ID and chain name or upload their own protein chain structures. Another database is from a benchmark of flexible molecules with conformations. This benchmark is the Database of Macromolecular Movements (MolMovDB), which contains a diverse set of molecules that display conformational changes in proteins and other macromolecules (http://www.molmovdb.org/), also including the intermediate morphs. The original benchmark has the total 2,695 PDB files that are classified into 214 groups. We typically choose around 30 groups with large conformational changes from MolMovDB for 3DMolNavi. The user selects a molecule from MolMovDB and 3DMolNavi computes the similarity measure for all molecules in the database, where the button “Link to MolMovDB” links to the website in the MolMovDB database to see how deformation of the query molecule works.

To compare the effectiveness of our method, we compare IDSS with some known 3D shape retrieval methods: D2, geodesic distance (GD), Shape Distribution (SD), Spherical Harmonic Descriptor (SHD), and Solid Angle Histogram (SAH) in terms of the performance in retrieving similar shapes. 

· Shape Distribution (SD) [4,6,22] represents the shape descriptor as a probability distribution sampled from a shape function measuring the geometric properties of a 3D model. Here, we use the *D*2 shape measure for the triangle surface of a molecule.

· Spherical Harmonic Descriptor (SHD) [4-6,19,20,28] is a rotation invariant shape descriptor based on spherical harmonics.

· Solid Angle Histogram (SAH) [4,6,29,30] measures the concavity and the convexity of a molecular surface. Histograms are computed based on a complete partitioning of the 3D space into disjoint cells which correspond to the bins of the histograms.

We use standard evaluation procedures from information retrieval, namely *precision-recall* curves, for evaluating the various shape retrieval methods. Precision-recall curves describe the relationship between precision and recall for an information retrieval method. A perfect retrieval retrieves all relevant models consistently at each recall level, producing a horizontal line at a precision = 1.0.

**Figure 4:**
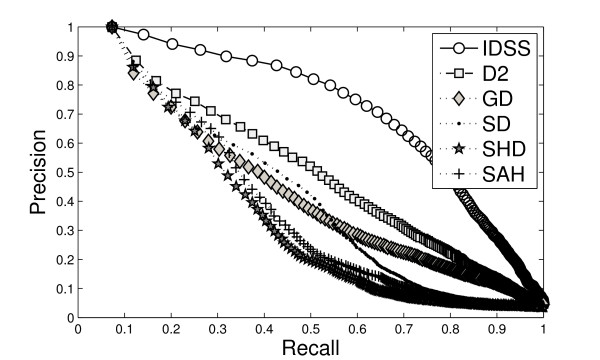
Precision-recall curves compared to other descriptor methods for the MolMovDB database.

Figure 4 shows the precision-recall curves for a subset of the MolMovDB database. For precision-recall plots, the precision for each molecular group is averaged using linear interpolation over the recall range. Furthermore, we also provide performance numbers besides the precision-recall curves. Table 1 shows the area under curve (AUC) of the recall-precision curve for MolMovDB. The AUC is a measurement showing how good the descriptor is. The higher AUC is, the greater the descriptor can achieve. The results show that the IDSS method performs better than other descriptors for the MolMovDB database with vastly different conformation variations.

## Discussion

3DMolNavi can be applied to several future applications. One is to search molecular databases for computer-aided drug design, which aims to identify compounds that are complementary to the site in molecular shape. Some softwares that treat molecules as rigid shapes have been proposed for this purpose. For instance, Zauhar et al. [25] developed a tool for searching the NCI database and the Tripos fragment database. Ballester et al. [2] implemented a software for retrieving several compound databases, including the Vendor Database and an independent benchmark from DrugBank. However the molecular databases may include some information about the flexibility of the molecule with its possible conformations, the traditional methods can not capture the shape similarity of flexible molecules well. The presented 3DMolNavi tool may directly replace the existing rigid methods for search the molecular databases.

Another application is protein structure retrieval. There have been many protein structural comparison methods presented by computing the similarity scores, but most of them are based on protein structure alignment, such as DALI and CE. In contrast, 3DMolNavi presented in this paper can be applied to a search for similar protein structures. The main advantage is that the shape-based protein searching method does not produce any alignment between two proteins.

The third application may be in cryo-electron microscopy (cryo-EM), where sheer shape comparison is important for example in discovery of high resolution structural homologues from cryo-EM maps. 3DMolNavi can overcome the different resolutions by considering both the geometrical shape and flexibility. For more explanation of applications, the reader can refer to [9,10].

**Table 1:** The AUC measures evaluated on the MolMovDB database with different descriptors

	
**Methods**	**AUC**
IDSS	0.619626
D2	0.448564
GD	0.340597
SD	0.390417
SHD	0.316265
SAH	0.348913

The main advantage of our method is to deal with flexible molecular shape comparison with large deformations. However, our method has no distinct advantage for some small molecules with little deformation, in contrast to previous works [2,3]. It is expected that the presented IDSS method can be considered as an alternative and complementary tool for the existing methods for protein structure comparison and rigid molecular shape comparison.

## Conclusions

The new software, named 3DMolNavi, has been developed for retrieval and navigation during flexible molecular shape comparison. 3DMolNavi is based on the IDSS histogram for fast retrieving molecules that are similar to a query molecule, and uses dimensionality reduction to navigate the retrieved results in 2D and 3D spaces. We tested 3DMolNavi in the Database of CATH and MolMovDB. Compared to other shape descriptors, our tool achieves better performance and retrieval results for different classes of flexible molecules. The advantages of 3DMolNavi, over other existing softwares, are to integrate retrieval for flexible molecular shape comparison and enhance navigation for user’s interaction.

## Availability and requirements

**Software name:** 3DMolNavi

**Software homepage:**https://engineering.purdue.edu/PRECISE/3dmolnavi/index.html

**Operating system(s):** Windows system is tested

**Web browser:** Mozilla Firefox 7, Chrome 14 and Internet Explorer 8 are tested

**Programming language:** Matlab and Flex (Action Script 3)

**Other requirements:** No

**License:** No

## Abbreviations

IDSS, Inner Distance Shape Signature; MDS, Multidimensional Scaling; MolMovDB, Database of Macromolecular Movements; AUC, area under curve; RMSD, root-mean-square deviation; SD, Shape Distribution; SHD, Spherical Harmonic Descriptor; SAH, Solid Angle Histogram.

## Competing interests

The authors declare that they have no competing interests.

## Author’s contributions

YL generated the original idea, executed the research and wrote the manuscript. MW implemented the idea. JP and KR supported and participated in the research. All authors read and approved the final manuscript.
